# Main characteristics of dermatoglypics associated with schizophrenia and its clinical subtypes

**DOI:** 10.1371/journal.pone.0252831

**Published:** 2021-06-10

**Authors:** Oyunchimeg Norovsambuu, Altansukh Tsend-Ayush, Nasantsengel Lkhagvasuren, Sarantuya Jav

**Affiliations:** 1 National Center of Mental Health, Ministry of Health, Ulaanbaatar, Mongolia; 2 Mongolian National University of Medical Sciences, Ulaanbaatar, Mongolia; University of Toronto, CANADA

## Abstract

Dermatoglypic patterns are extensively investigated to apply in disease-related risk assessment due to an obvious association between morphological and genetic characteristics. In the current study, we aimed to determine whether the fingerprint and palmar patterns vary between case population with schizophrenia and general population. A cross sectional study was conducted in people diagnosed with schizophrenia (cases) and a control population between 2016 and 2019. In this study, 252 people were participated. Ink and paper method was used to evaluate the difference of fingerprints palm prints between patients with schizophrenia and participants in control group.93 participants were analyzed in schizophrenic group and 142 participants were investigated in the control group. The percentage of arches on the right ring finger was significantly different between the schizophrenic patient group and control group (*p = 0*.*011*). The whorl pattern type (U-W-U-W-W-W-W-U-W-U) was dominantly observed in both of the schizophrenic patient group and control group. A-B ridge count in schizophrenic patient group and control group produced a markedly significant difference (*p<0*.*05*). Interestingly, a strong significant difference was produced in comparing of A-B ridge count in catatonic schizophrenia group with residual schizophrenia group (*p<0*.*005*). In comparison, index of pattern intensity in control group was slightly higher than that in schizophrenic patient group. Taking together, these results showed that the dermatoglypic characteristics might be a valuable tool to describe the nature of schizophrenia and its clinical subtypes and further studies are needed in clinical application.

## Introduction

Schizophrenia is a mental illness characterized by a complex symptoms including hallucinations, delusions, disorganized speech, extremely disordered behavior and impaired cognitive ability [1]. In the diagnosis of Schizophrenia, the Diagnostic and Interview for Psychosis (DIP) and Diagnostic and Statistical Manual of Mental Disorders, Fifth Edition (DSM-5) are currently used to describe a range of cognitive, behavioral, and emotional symptoms that make a diagnosis of Schizophrenia [2]. So far, the diagnostic and treatment methods of schizophrenia are obviously improved by specialists and investigators in comparison with those in past time [3,4]. No simple physical and laboratory tests are available for diagnosis of schizophrenia. Moreover, no physically simple risk assessment methods are applied to suspect the schizophrenia for clinical cases. Therefore, schizophrenia has still been attracted an attention to investigate in many fields of biological and medical sciences to understand its nature and discover a novel diagnostic and risk assessment method.

Dermatoglypics describes a study of epidermal ridges and their drawing on the fingers and palmar area. The epidermal ridges and their drawings are developed early in life and never changed throughout the individual’s lifetime [5]. It is well-known that the drawing of ridges on finger and palmar areas varies in people and it depends on individual’s genetic profile [6]. In addition, dermatoglypic patterns are investigated to apply in disease-related risk assessment due to an obvious association between morphological and genetic characteristics [7]. Therefore, the variability of the finger and palmar ridges properties have been applied as an indicator of evaluating the risk for some multifactorial disorders such as arterial hypertension [8], cardiovascular diseases [9], allergy [10], cancer [11,12] and schizophrenia [13,14]. Many studies have provided evidence that the individual changes on the finger and palmar ridges are associated with multifactorial diseases [15]. Hence, dermatoglypics has been recommended as a routine component of risk assessment for these diseases.

In medical genetics, dermatoglyphics has been extensively investigated to discover a new feature in finger and palmar ridges of patients with schizophrenia due to unchangeable nature of fingerprints and palmar ridges of individual people [16,17]. There are many studies conducted to reveal the dermatoglypic features related with schizophrenia in recent years. It reported that percentage of total loops in patients with schizophrenia was increased while percentage of whorl and arches was decreased in comparison with general population [14,18]. Also, it was reported that total finger ridge count was reduced in patients with schizophrenia [13,19]. Moreover, value of “atd” angle is observed in higher as compared in general population [20].

There are some studies found on relationship between dermatoglypic characteristics and schizophrenic clinical subtypes. Jhingan et al reported that atd angle was significantly different in control and catatonic schizophrenia group, and arch pattern was also dominantly observed in catatonic schizophrenia group. So far, it still needs more comprehensive studies to describe the dermatoglypic nature for schizophrenia clinical subtypes, including simple, reduced, paranoid and catatonic schizophrenia.

In the current study, we aimed to determine whether the fingerprint and palmar patterns vary between people with schizophrenia and general population, and reveal the dermatoglypic nature of schizophrenia clinical subtypes. For this purpose, the patients with schizophrenia was diagnosed by using diagnostic criteria and tests, and classified into 4 groups based on their clinical types. Healthy population was chosen in accordance with the inclusion criteria. The study covered differences of all fingerprint pattern types, total finger ridge count, A-B ridge count, and indices related with fingerprint pattern types and triradii-related angles between schizophrenic patient group and control group.

## Materials and methods

### Study design

A cross sectional study was conducted in people with schizophrenia and control population in the period between 2016 and 2019. In the current study, 252 people were participated. Among them, 102 people were participated in the patient group and 150 people were participated in the control group. Informed written consent to participate was obtained from all participants and legal guardians.

### Sample size

A total of 235 participants, including 93 patients with schizophrenia and 142 people without mental disorders, were chosen to participate in this study. The participants were in a range of 20–64 years old and both sexes. The people without mental disorders were categorized as control group.

### Ethical approvals

Ethical clearance was obtained from Medical Ethical Review Board of the Mongolian University of Medical Sciences (Approval number: №2016/3-2016-22).

### Study settings

#### Patients with schizophrenia

The patients with schizophrenia were selected from National Center of Mental Health and diagnosed by psychiatrists according to Diagnostic and Interview for Psychosis (DIP-DM). All medical history and records of the patients were obtained. In the study, the schizophrenia was categorized according to international classification of diseases 10 (ICD-10). The legal guardians consent was taken for the patients with schizophrenia to participate in the study.

#### Inclusion criteria

In our study, several inclusion criteria were applied to select the patients with schizophrenia. Participants should be a Mongolian, given informed consent from legal guardian, have ability to understand and respond for questionnaire.

#### Exclusion criteria

Foreign national was not allowed to participate in the study. Schizophrenic patients with burns or wounds, and any deformities on the palms and those who had other genetically linked conditions except schizophrenia were excluded. Also, those who did not give consent were excluded. In the patient group, a total of 102 participants were included to this study. Among them, 93 were participated and 9 were excluded. One who had a history of cutting a finger off was excluded. Another one was excluded due to finger deformities. 7 participants who didn’t give consent for the study were also excluded.

#### Control groups

The control group consisted of healthy volunteers from the staff of National Center of Mental Health and students of Mongolian National University of Medical Sciences. The participants in the control group were selected randomly by researchers. A mental health medical doctor obtained a comprehensive history and examination on their health. All the participants were confirmed that they had not any mental disorders and no family history of schizophrenia. All the participants gave their consent to participate in the study.

#### Inclusion criteria

Healthy volunteers were participated in this study. Participants should be a Mongolian and gave informed consent for the study.

#### Exclusion criteria

The participants were excluded if they had a past history of schizophrenia. Foreign national was not allowed to participate in the study. Those people with burns or wounds, and any deformities on the palms and those who had other genetically linked conditions were excluded. Also, those who did not give consent were excluded. Our study covered a total of 150 participants in the control group and 8 of them were excluded due to the following reasons. A participant was excluded, who had a history of cutting a finger off. 7 participants who didn’t give consent for the study were also excluded from the control group.

#### Diagnostic interview for psychoses

International classification of diseases-10 (ICD-10) was used to record patient’s medical history and make a clinical diagnosis of patients with schizophrenia. Briefly, participant was interviewed in a face to face interaction to collect answers according to the criteria, and then do a clinical examination. Firstly, a role in social work, working status, family history on psychological and physical illness and life history of individual development were evaluated in the socio-demographic interview (around 45 min). Subsequently, patient’s medical history was recorded by psychiatrists according to the DIP criteria, Version 4.1. To generate psychiatric diagnoses, Operational Criteria Checklist (OPCRIT) was used.

### Examination for fingerprints and palm prints

#### Materials

Print ink, a rubber roller, an inking slab (a thick glass sheet which was fixed on a wooden support), an A4 size white sheet, a pressure pad which was made of rubber foam, cotton puffs, a scale, a pencil and a pen. The fingerprints were taken on an A4 size paper by the rolling finger technique.

#### Techniques to obtain fingerprint and palm prints

In this study, ink and paper method [21] was used to evaluate the difference of fingerprints palm prints between patients with schizophrenia and participants in control group. Briefly, all participants were required to wash their hands to remove dirt before taking fingerprints and palmar dermatoglypic patterns. A small amount of printer ink was applied on glass board with a roller. Then, the palm was smeared uniformly with inked roller to cover the whole area of the palm and fingers for the examination. Subsequently, the palm and fingers were successively rolled over a white paper by using some pressure and obtained the finger prints and palm prints. The finger prints were obtained with rotation of fingers to take complete imaging of fingertip. Finally, the finger and palmar prints were visualized by a canon visualizer (Canon RE-455X Visualizer, USA) to obtain the data from finger and palm.

#### Fingerprint pattern evaluation

**Fig 1:**
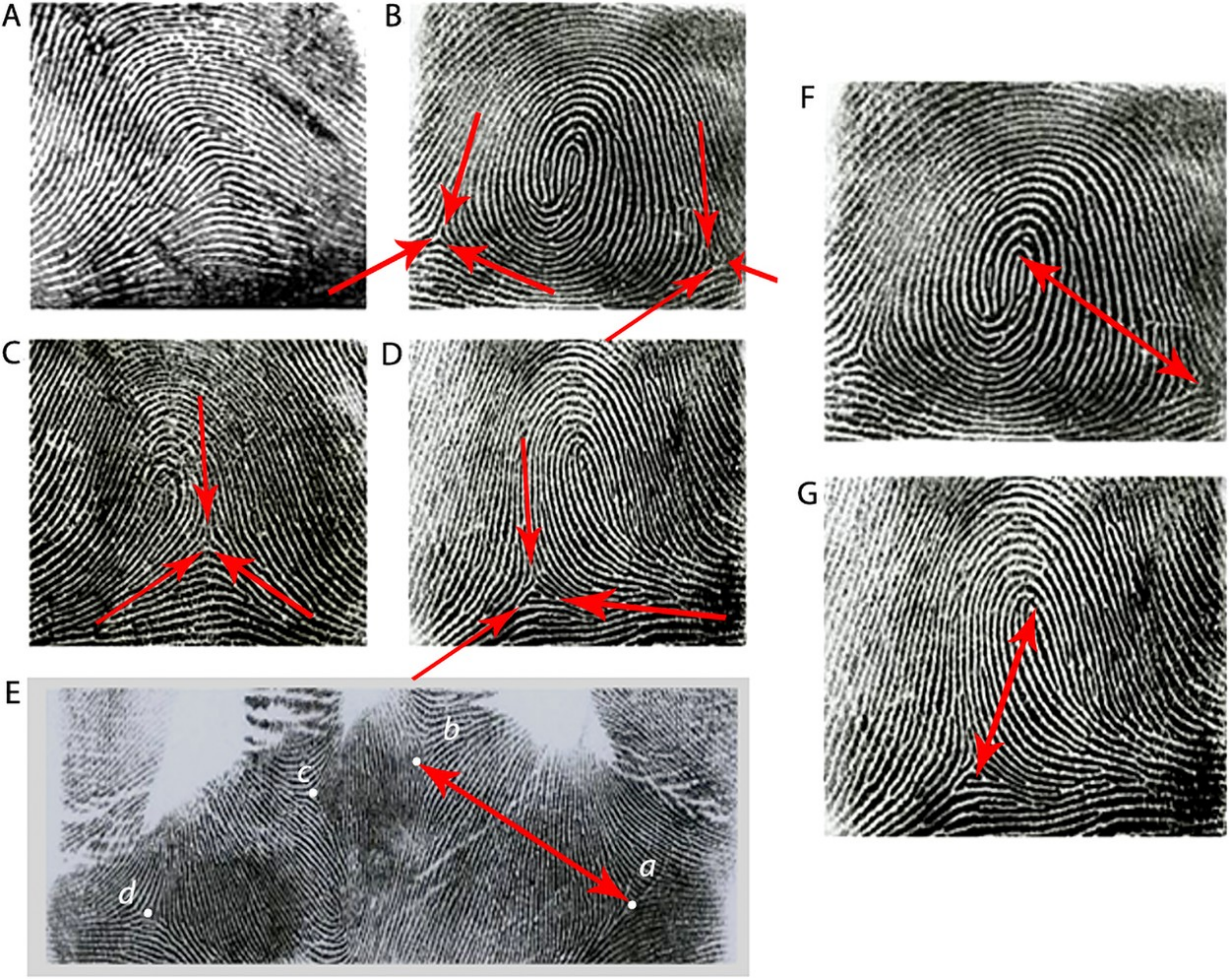
Fingerprint pattern types: A. Arch, B. Whorl, C. Radial loop, D. Ulnar loop, E. A-B ridge count, F. Finger ridge count in whorl pattern, G. Finger ridge count in loop pattern.

The finger fingerprint patterns were classified into 4 types: ulnar loop (UL), radial loop (RL), arch (A) and whorl (W) Fig 1. Any pattern that could not be classified was marked as undefined. Patterns that were in severely scared areas where details could not be observed or where amputation had occurred were marked as missing.

#### Finger ridge count

Finger ridge count was defined as the number of ridges which intersect or touch a straight line drawn from the central point of a triradius to the center or core of the adjacent pattern Fig 1. Two ridges that result from a bifurcation of a single epidermal ridge and both cross the straight line are counted. Any ridges that were close to the straight line without touching it were excluded. Loop patterns have one ridge count while whorls usually have two ridge counts. Arches and other similar configurations that are not true patterns have a zero ridge count. When there was a missing finger on one hand, the ridge count on the corresponding finger of the other hand was inserted based on the considerable symmetry for this trait.

#### Total ridge count

The sum of the largest ridge count on all ten fingers was defined as total ridge count (TRC).

#### A-B ridge count

On the palm, the number of ridges that crossed a straight line connecting triradii “A” and “B” was defined as the A-B ridge count (A-B RC) Fig 1.

#### Pattern intensity

The pattern intensity index (PII) is defined as a combined value of whorl and loop patterns from fingers. PII was calculated using the following formula (1):

$$PII=\frac{2\;x\;\%\;of\;whorl+\%\;of\;loop}{10}$$ (1)

#### Dankmeijer index

The Dankmeijer index (DI) is defined as the relative percentage of arch pattern to whorl pattern from fingers. It was calculated using the formula (2):

$$\text{DI}=\frac{\%\;\text{of}\;\text{arch}}{\%\;\text{o}\;\text{whorl}\;}\text{x}\;100$$ (2)

#### Furuhata index

The Furuhata index (FI) is defined as the relative percentage of whorl pattern to loop pattern from fingers. FI was calculated using the formula (3):

$$FI=\frac{\%\;of\;whorl\;}{\%\;of\;loop\;}\;x\;100$$ (3)

#### “atd”, “dat” and “tda” angle evaluation

Triradii were termed according to letters of the English alphabet. The “*atd”* angle is the angle between two straight lines joining the *a*-triradius and the *d*-triradius, under the little finger, with a point *t*, on the lower outer portion of the palm, approximately under the ring finger. The “*dat”* angle is the angle between two straight lines joining the *d*-triradius and the *t*-triradius, with a point *a*-triradius. The “*tda”* angle is the angle between two straight lines joining the *t*-triradius and the *a*-triradius, with a point *a*-triradius. The palmar angles were measured with a protractor angle ruler.

### Statistical analysis

All data were analyzed using GraphPad Prism 5 and SPSS^®^ Version 22. The social-demographic clinical data were evaluated by using descriptive statistics. All data were presented with mean and standard deviation (SD). The frequencies and differences of fingerprints and palm prints within two groups were assessed by using chi-square test. P value of < 0.05 was considered statistically significant.

## Results

### Characteristics of the screened population

In the current study, a total of 235 participants were analyzed to evaluate the fingerprint and palm prints characteristics in the schizophrenic patient group and control group (people without mental disorders). Among them, 93 participants were analyzed in schizophrenic group and 142 participants were investigated in the control group. The demographic data were presented in Table 1.

**Table 1:** Demographic characteristics of screen population.

Category	Patient group (Schizophrenia) (n = 93)	Control group (Comparative) (n = 142)	Mean ± SD	Total number (percentage)
**Age**	40.1 ± 11.5	39.9 ± 12.6	40 ± 12.19	
**Sex**				
Male	45 (48.4%)	69 (48.6%)	-	114 (48.5%)
Female	48 (51.6%)	73 (51.4%)	-	121 (51.5%)
**Diagnoses or clinical types**				
Paranoid schizophrenia (F20.0)	49 (52.7%)	-	-	
Catatonic schizophrenia (F20.2)	6 (6.5%)	-	-	
Residual schizophrenia (F20.5)	28 (30.1%)	-	-	
Simple schizophrenia (F20.6)	10 (10.7%)	-	-	

According to Table 1, the mean age of participants in both groups was 40 ± 12.19.48.4% of participants were male and 51.6% of the participants were female in the schizophrenic patient group whereas 48.6% of participants were male and 51.4% of the participants were female in the control group. The participants in the schizophrenic patient group were categorized by using international classification of diseases 10 (ICD-10). In this study, 4 types of schizophrenia, including paranoid, catatonic, residual and simple schizophrenia, were diagnosed and analyzed to reveal the differences of dermatoglypic properties in schizophrenic patients.

### Evaluation of fingerprint pattern types

The fingerprint pattern types were extensively investigated by evaluating the numbers and percentage of each pattern type at each finger of right and left hands. Data were shown in Table 2. In comparison, no statistical significant differences in the percentages of whorl and loop pattern types in each finger was observed in the schizophrenic patient group and control group (*p>0*.*05*). Interestingly, only the percentage of arches on the right ring finger was significantly different in the schizophrenic patient group and control group (*p = 0*.*011*), suggesting that arch pattern type in right ring finger might be a dermatoglypic marker for schizophrenic patients. The mean percentage of whorl, loop and arch in all 10 finger was 49.7%, 44.8% and 5.5 in the schizophrenic patient group. In the control group, the mean percentage of whorl, loop and arch in all 10 fingers was evaluated 50.14%, 46.26% and 3.6%, respectively. There was no significant difference found between the mean percentage of whorl, loop and arch in the two groups.

**Table 2:** Numbers and percentage of fingerprint pattern types in right and left hands.

Fingerprint pattern types	Study groups	Sex	Left hand					Right hand				
			Little	Ring	Middle	Index	Thumb	Thumb	Index	Middle	Ring	Little
**Whorl**	Patient	M	12	30	17	21	21	29	26	22	32	18
		F	20	24	15	26	29	31	27	17	26	19
		**%**	**34.4**	**58.0**	**34.4**	**50.5**	**53.8**	**64.5**	**57.0**	**41.9**	**62.4**	**39.8**
	Control	M	18	42	34	42	35	46	45	33	50	26
		F	20	45	29	31	44	45	37	21	52	17
		**%**	**26.8**	**61.3**	**44.4**	**51.5**	**55.7**	**64.1**	**57.7**	**38.0**	**71.8**	**30.3**
**U loop**	Patient	M	33	15	27	18	24	15	10	22	12	27
		F	27	22	30	11	16	16	12	27	17	28
		**%**	**64.5**	**39.8**	**61.3**	**31.2**	**43.0**	**33.3**	**23.7**	**52.7**	**31.2**	**59.1**
	Control	M	51	26	29	19	30	19	11	32	19	43
		F	52	28	40	30	27	27	28	49	20	54
		**%**	**72.5**	**38.0**	**48.6**	**34.5**	**40.1**	**32.4**	**27.5**	**57.0**	**27.5**	**68.3**
**R loop**	Patient	M	-	-	1	1	-	-	2	-	-	-
		F	1	-	0	3	-	-	0	-	-	-
		**%**	**1.1**	**-**	**1.1**	**4.3**	**-**	**-**	**2.2**	**-**	**-**	**-**
	Control	M	-	1	1	4	0	-	7	-	-	-
		F	-	0	1	6	2	-	1	-	-	-
		**%**	**-**	**0.7**	**1.4**	**7.0**	**1.4**	**-**	**5.6**	**-**	**-**	**-**
**Arch**	Patient	M	-	0	0	5	0	1	7	1	1	0
		F	-	2	3	8	3	1	9	4	5	1
		**%**	**-**	**2.2**	**3.2**	**14.0**	**3.2**	**2.2**	**17.1**	**5.4**	6.4*	**1.1**
	Control	M	0	-	5	4	4	4	6	4	0	0
		F	1	-	3	6	0	1	7	3	1	2
		**%**	**0.7**	**-**	**5.6**	**7.0**	**2.8**	**3.5**	**9.2**	**5.0**	0.7*	**1.4**

**Expressions**: M-Male; F-Female; (*p<0.*011*).

As shown in Table 2, the whorl pattern type (U-W-U-W-W-W-W-U-W-U) was dominantly observed in both of the schizophrenic patient group and control group. Also, the loop pattern type was found as the second most common pattern type in the study groups. Hence, we have obtained the common fingerprint distribution in 10 fingers by using Galton formula (From little finger in left hand to little in right hand) in the two groups. It was the same as U-W-U-W-W-W-W-U-W-U in the schizophrenic patient group and control group.

In the current study, the fingerprint pattern types were investigated with schizophrenia clinical types for evaluation. As compared to each schizophrenia clinical types, there were no statistically significant difference produced *(p>0*.*05)* and the data were depicted in Table 3. Interestingly, it is notable that the patients with catatonic schizophrenia types had more whorl and less radial loop fingerprint types.

**Table 3:** Numbers and percentage of fingerprint pattern types by schizophrenia clinical types.

Schizophrenia clinical types	Fingerprint types	Fingerprint pattern percentage (%)									
		Left hand					Right hand				
		Little	Ring	Middle	Index	Thumb	Thumb	Index	Middle	Ring	Little
Paranoid schizophrenia (F20.0) (n = 49)	Whorl	38.8	59.2	32.7	47.0	53.0	59.2	55.2	38.8	65.3	40.9
	Ulnar loop	61.2	36.7	61.2	36.7	42.9	38.8	26.5	57.1	26.5	57.1
	Radial loop	-	-	2.0	4.1	-	-	2.0	-	-	-
	Arch	-	4.1	4.1	12.2	4.1	2.0	16.3	4.1	8.2	2.0
	**Distribution**	U	W	U	W	W	W	W	U	W	U
Catatonic schizophrenia (F20.2) (n = 6)	Whorl	50.0	83.3	50.0	66.7	83.3	83.3	83.3	66.6	83.3	50.0
	Ulnar loop	50.0	16.7	50.0	-	16.7	16.7	-	16.7	-	50.0
	Radial loop	-	-	-	-	-	-	-	-	-	-
	Arch	-	-	-	33.3	-	-	16.7	16.7	16.7	-
	**Distribution**	W/U	W	W/U	W	W	W	W	W	W	W/U
Residual schizophrenia (F20.5) (n = 28)	Whorl	28.6	53.6	35.7	50.0	53.6	71.4	57.2	42.9	53.6	35.7
	Ulnar loop	71.4	46.4	60.7	28.5	42.8	25.0	21.4	50.0	42.8	64.3
	Radial loop	-	-	-	3.6	-	-	-	-	-	-
	Arch	-	-	3.6	17.9	3.6	3.6	21.4	7.1	3.6	-
	**Distribution**	U	W	U	W	W	W	W	U	W	U
Simple schizophrenia (F20.6) (n = 10)	Whorl	20.0	50.0	30.0	60.0	40.0	60.0	50.0	40.0	60.0	40.0
	Ulnar loop	70.0	50.0	70.0	30.0	60.0	40.0	30.0	60.0	40.0	60.0
	Radial loop	-	-	-	10.0	-	-	10.0	-	-	-
	Arch	10.0	-	-	-	-	-	10.0	-	-	-
	**Distribution**	U	W/U	U	W	U	W	W	U	W	U

The common fingerprint distribution was also described in the evaluation of fingerprint types with schizophrenia clinical types. The difference between the fingerprint distributions in groups of schizophrenia clinical types was found in catatonic and simple schizophrenia clinical types. The more whorl fingerprint distribution was found in catatonic type while the simple schizophrenia had more loop fingerprint pattern types.

### Total ridge count and finger ridge count

Firstly, the total finger ridge count was evaluated in the schizophrenic patient group and control group. The mean value of total finger ridge count in schizophrenic patient group was 136.7 ± 43.9 whereas it was 139.5 ± 38.8 in control group. In comparison, no statistically significant difference was found between the total finger ridge counts in the two groups (*p>0*.*05*). The data were displayed in Table 4.

**Table 4:** Mean of total finger ridge count.

Study groups	Sex	Sample count	Mean ± Stdev.	P value
**Patient group** (Schizophrenia) n = 93	Male	45	145.3± 39.18	*p>0*.*05*
	Female	48	128.6± 46.94	
	**Total**	93	136.7 ± 43.9	
**Control group** (Comparative) n = 142	Male	69	147.9± 41.28	
	Female	73	131.6± 34.82	
	**Total**	142	139.5 ± 38.8	

Subsequently, the total ridge count expressed by schizophrenia clinical subtypes was investigated in this study. There was no significant difference produced between the total finger count in the schizophrenic clinical types (p>0.05). The data were presented and expressed with *p* value in Table 5.

**Table 5:** Mean of total finger ridge count by schizophrenia clinical types.

Schizophrenia clinical types	Sex	Sample count	Mean ± Stdev.	P value
Paranoid schizophrenia (F20.0)	Male	n = 20	152.2 ± 38.7	PvsC; *p = 0*.*382* PvsR; *p* = 0.781 PvsS; *p* = 0.977 CvsR; *p* = 0.259 CvsS; *p* = 0.495 RvsS; *p* = 0.866
	Female	n = 29	125.5 ± 48.7	
	**Total**	n = 49	136.46 ± 46.41	
Catatonic schizophrenia (F20.2)	Male	n = 2	129.0 ± 120.2	
	Female	n = 4	167.7 ± 34.6	
	**Total**	n = 6	154.83 ± 63.32	
Residual schizophrenia (F20.5)	Male	n = 18	139.4 ± 30.9	
	Female	n = 10	123.2 ± 42.0	
	**Total**	n = 28	133.64 ± 35.42	
Simple schizophrenia (F20.6)	Male	n = 5	145.6 ± 39.4	
	Female	n = 5	126.4 ± 52.1	
	**Total**	n = 10	136 ± 44.72	

**Expressions**: P-Paranoid; C-Catatonic; R-Residual; S-Simple.

**Fig 2:**
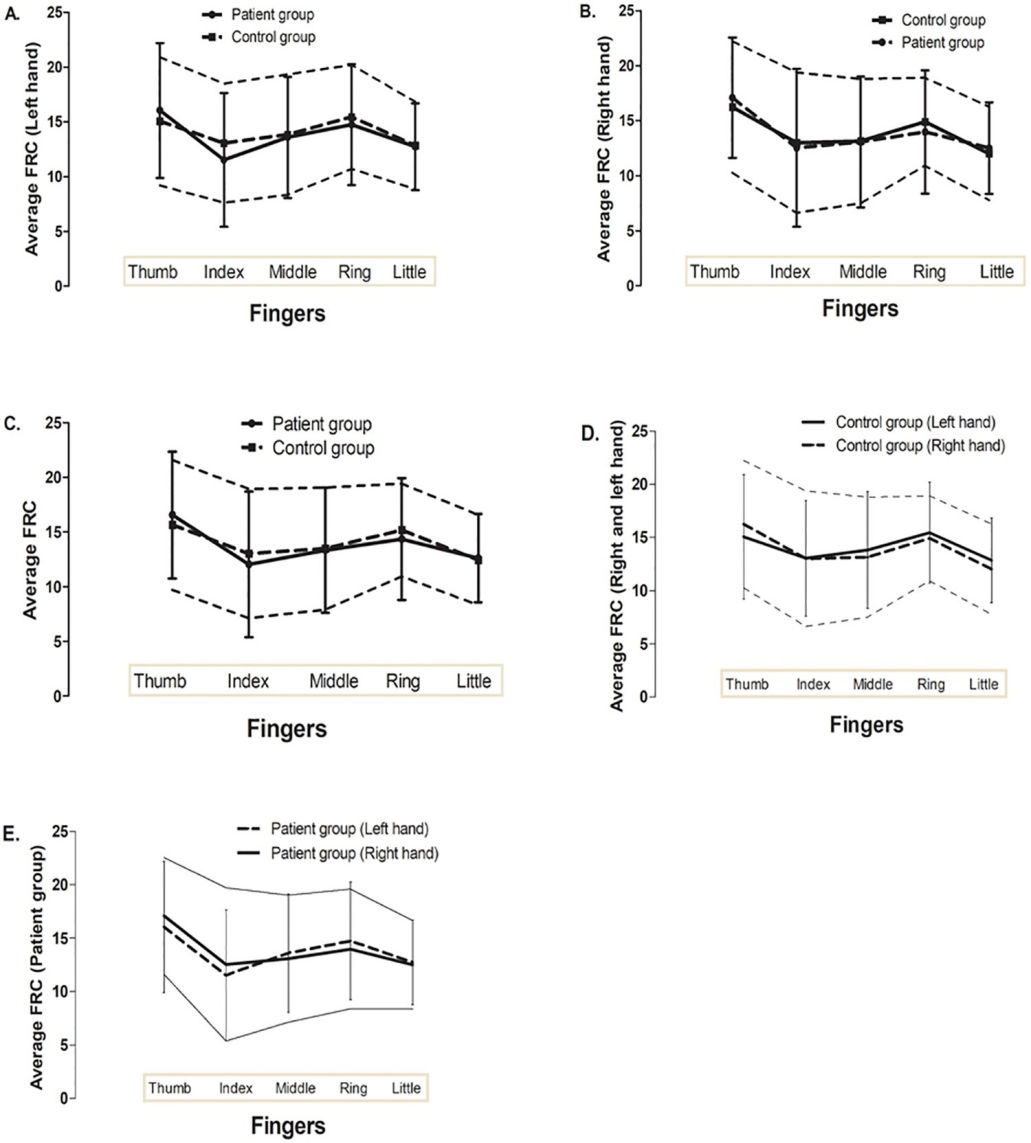
Pattern of average finger ridge count by each finger A. Average FRC (Left hand) B. Average FRC (Right hand) C. Average FRC in patient and control group D. Average FRC in control group (Left and right hand) E. Average FRC in patient group (Left and right hand).

In the current study, we have drawn a pattern that would express the changes of each finger ridge count by using the average finger ridge count. As shown in Fig 2, the higher finger ridge count was found in the thumb of right and left hands while the lowest finger ridge count was observed in the index and little fingers of two hands. The average finger ridge counts of thumb, index finger, middle finger, ring finger and little finger were 16.56 ± 5.8, 12.04 ± 6.66, 13.33 ± 5.73, 14.36 ± 5.56 and 12.32 ± 4.04 in schizophrenic patient group, respectively. Regards with control group, the average finger ridge counts of thumb, index finger, middle finger, ring finger and little finger were 15.65 ± 5.93, 13.02 ± 5.91, 13.48 ± 5.57, 15.18 ± 4.24 and 12.43 ± 4.12, respectively. No statistically significant difference was observed between finger ridge counts in schizophrenic patient group and control group.

### A-B ridge count

A-B ridge count was evaluated in the study groups and data were expressed with mean with standard deviation in Table 6. In comparison, A-B ridge count in schizophrenic patient group and control group produced a markedly significant difference (*p<0*.*05*), indicating that a reduced A-B ridge count is important dermatoglypic characteristic for schizophrenic patient. Such a significant difference between A-B ridge count was also observed between the females in schizophrenic patient group and control group (*p<0*.*01*).

**Table 6:** Mean of A-B ridge count.

Study groups	Sex	Sample count	Right/Left hand	Mean ± Stdev	P value
**Patient group** (Schizophrenia) n = 93	Male	n = 45	Right	35.44± 6.86	*p = 0*.*02*
			Left	35.24 ± 6.3	
	Female	n = 48	Right	33.79± 7.13	
			Left	34.6 ± 6.56	
	**Total**	n = 93		69.5± 11.7	
**Control group** (Comparative) n = 142	Male	n = 69	Right	36.36± 5.83	
			Left	36.42 ± 5.7	
	Female	n = 73	Right	35.32± 5.96	
			Left	37.56 ± 5.35	
	**Total**	n = 142		72.8± 9.8	

In schizophrenic patient group, A-B ridge count was also evaluated to reveal the different properties between clinical types of schizophrenia. As shown in Table 7, no statistically significant differences were found between the clinical types of P vs. C, P vs. R, P vs. S, C vs. S and R vs. S. Interestingly, a strong significant difference was produced in comparing of A-B ridge count in catatonic schizophrenia group with residual schizophrenia group (*p<0*.*005*). According to the result, it can be suggested that A-B ridge count of the patients with catatonic schizophrenia is the highest.

**Table 7:** Mean of A-B ridge count by schizophrenia clinical types.

Schizophrenia clinical types	Sex	Right/Left hand	Sample count	Mean ± Stdev.	P value
Paranoid schizophrenia (F20.0)	Male	Right	n = 20	37.6 ± 5.7	P vs. C*p = 0*.*09* P vs. R *p = 0*.*07* P vs. S *p = 0*.*75* C vs. R *p = 0*.005* C vs. S *p = 0*.*06* R vs. S *p = 0*.*33*
		Left		36.6 ± 5.2	
	Female	Right	n = 29	33.4 ± 7.8	
		Left		34.5 ± 7.1	
	**Total**		n = 49	70.5 ± 12.3	
Catatonic schizophrenia (F20.2)	Male	Right	n = 2	46.0 ± 4.2	
		Left		45.5 ± 2.1	
	Female	Right	n = 4	38.7 ± 2.8	
		Left		34.7 ± 4.6	
	**Total**		n = 6	79.5 ± 10.9	
Residual schizophrenia (F20.5)	Male	Right	n = 18	32.3 ± 6.6	
		Left		32.4 ± 6.6	
	Female	Right	n = 10	33.4 ± 5.9	
		Left		33.8 ± 6.7	
	**Total**		n = 28	65.6 ± 10.2	
Simple schizophrenia (F20.6)	Male	Right	n = 5	33.8 ± 6.4	
		Left		35.6 ± 4.3	
	Female	Right	n = 5	32.8 ± 7.5	
		Left		36.2 ± 4.3	
	**Total**		n = 10	69.2 ± 9.3	

***Expressions***: P-Paranoid; C-Catatonic; R-Residual; S-Simple (*—significant difference).

### Indices of fingerprint patterns

In the current study, the indices of pattern intensity, dankmeijer and furuhata were evaluated to reveal the common frequency of fingerprints in patient groups and control groups. In comparison, index of pattern intensity in control group was slightly higher than that in schizophrenic patient group. The slight difference of pattern intensity index was also observed in sex categories of two study groups. Interestingly, evidently higher pattern intensity index was detected in catatonic schizophrenia as compared to other schizophrenia in the patient group. Index of Dankmeijer was higher in schizophrenic patient group and lower in control group. Regards with the schizophrenic clinical types, the index in the simple schizophrenia was comparably lower in comparison with other clinical types. Furuhata index in the schizophrenic patient group was detected the same as that in the control group. In the control group, index of Furuhata in male population was relatively higher than that in female population. A highest index of Furuhata was observed in the catatonic schizophrenia patients and a lowest index of Furuhata was observed in the simple schizophrenia patients. The data describing the indices of fingerprint pattern was presented in Table 8.

**Table 8:** Indices of fingerprint patterns in study groups.

Study groups	Sex	Index of pattern intensity	Index of Dankmeijer	Index of Furuhata
**Patient group** (Schizophrenia) n = 93	Male	14.74	6.5	110.21
	Female	14.13	15.36	111.67
	**Total**	14.42	11.06	110.93
	**Indices by clinical types**			
	Paranoid	14.33	11.65	108.23
	Catatonic	16.16	11.9	322.78
	Residual	14.21	12.60	105.55
	Simple	14.3	4.44	84.9
**Control group** (Comparative) n = 142	Male	14.99	7.24	127.18
	Female	14.34	7.06	93.4
	**Total**	14.65	7.18	108.38

### Evaluation on “atd”, “tda” and“dat” angles

The palmar dermatoglypic patterns were analyzed with evaluating the “atd”, “tda” and “dat” angles to reveal the differences between the schizophrenic patient group and control group. As displayed in Table 9, the mean “atd” angle in schizophrenic patient group and control group produced no statistically significant differences.

**Table 9:** Mean values of angles of atd, tda and dat in study groups.

Study groups	Right/Left hand	“atd” angle Mean ± Stdev	“tda” angle Mean ± Stdev	“dat” angle Mean ± Stdev
**Patient group** (Schizophrenia) n = 93	Right	42.4± 4.85	79.6± 4.51	59.4± 4.58
	Left	42.41± 4.56	79.8± 4.68	58.8± 4.63
	**Total**	42.16±4.74	79.7±4.59	59.1±4.6
**Control group** (Comparative) n = 142	Right	41.92± 4.63	79.7± 3.93	58.5± 5.15
	Left	42.34± 4.98	79.4± 3.88	61.9± 4.11
	**Total**	42.38±4.77	79.5±3.9	58.4±4.95
**Patient vs Control group P-value**		*p = 0*.*72*	*p = 0*.*72*	*p = 0*.*276*

In addition, the mean values of atd, tda and dat angles were analyzed in the subclinical types of schizophrenia. In comparison, no significant difference was found between the mean values of atd, tda and dat angles of all subclinical types of schizophrenia. As shown in Table 10, the mean value of left hand atd angle in paranoid schizophrenia was observed significantly higher as compared to that in paranoid (*p<0*.*03*) and residual schizophrenia (*p<0*.*02)*. Also, a strong difference of tda angle was found in left hand of catatonic schizophrenia patient in comparison with that of paranoid schizophrenia group (*p<0*.*003)*. The mean value of left hand dat angle in catatonic schizophrenia was significantly higher than that in simple schizophrenia (*p<0*.*02*).

**Table 10:** Mean values of angles of atd, tda and dat in schizophrenic clinical types.

Schizophrenia clinical types	Right/Left hand	“atd” angle Mean ± Stdev	“tda” angle Mean ± Stdev	“dat” angle Mean ± Stdev
Paranoid schizophrenia	Right	42.06± 4.46	79.53± 4.27	59.59± 4.91
	Left	41.79± 4.31	80.81± 3.84	58.34± 4.33
	**Total**	41.92±4.35	80.17±4.09	58.96±4.65
Catatonic schizophrenia	Right	45.6± 5.27	79.16± 4.16	58.6± 5.16
	Left	42.3± 2.58	75.5± 4.8	62± 4.28
	**Total**	44±4.32	77.3±4.69	60.3±4.84
Residual schizophrenia	Right	41.85± 4.9	79.85± 4.56	60± 4.03
	Left	40.89± 4.81	79.32± 5.19	59.8± 5.01
	**Total**	41.37±4.88	79.58±4.85	59.91±4.51
Simple schizophrenia	Right	43.7± 5.92	80.2± 6.17	57.4± 4.06
	Left	45.2± 5.69	78.9± 5.6	56.7± 4.16
	**Total**	44.45±5.7	79.5±5.8	57.05±4.01
**P-value**	Left	P vs S; *p* = *0*.03*	P vs C; *p = 0*.003*	C vs S; *p = 0*.02*
		R vs S; *p* = *0*.02*		
	Right	*p*>*0*.*05*	*p*>*0*.*05*	*p*>*0*.*05*
	**Total**	*p*>*0*.*05*	*p*>*0*.*05*	*p*>*0*.*05*

***Expressions***: P-Paranoid; C-Catatonic; R-Residual; S-Simple (*—significant difference).

## Discussion

Dermatoglypics is commonly used in medicine to describe the basic fingerprint and palmar pattern related to a disease, especially for multifactorial diseases [15]. Nowadays, fingerprint and palmprint patterns are applied as a simple marker to evaluate disease-related differences and suspect some disease with obscure etiology and mysterious pathogenesis. For instance, main indicator characteristics of dermatoglypic pattern have been investigated on some chromosomal diseases such as Down’s syndrome [22], Trisomy 18 [23] and Turner’s syndrome [24], and multifactorial diseases such as cancer, bronchial asthma, intestinal disorders and mental illness [12,25–27].

In the current study, we have investigated the primary difference of fingerprint pattern types, total finger ridge count, A-B ridge count, indices of fingerprint patterns and angles related to palmar triradius. Firstly, common finger pattern types were studied to differentiate their nature between the schizophrenic patient group and control group. The percentage of arches on the right ring finger was observed in different manner in the study groups, suggesting that arch pattern type in right ring finger might be a dermatoglypic marker for schizophrenic patients. The result was consistent with previously reported studies [14,28]. The mean percentage of whorl, loop and arch in all 10 fingers was not statistically different in our study groups.

It is well-known that the distribution of fingerprint patterns is different in each single finger. It can be divided into arch, loop and whorl in accordance with the fingerprint pattern types. The common fingerprint distribution in 10 fingers varies in different race and countries [29]. The universal distribution of fingerprint patterns in the world was ulnar loop (U-U-U-U-U) and whorl (W-W-W-W-W) [30,31]. In our study, the universal distribution of 10 finger pattern types was also evaluated in the study groups and subclinical groups. It was U-W-U-W-W and no difference was observed in the study groups. The universal distribution of fingerprint patterns in Mongolians was more consistent with that in Chinese and Thais population. Interestingly, the universal distribution in catatonic schizophrenia (WU-W-W/U-W-W-W-W-W-W-W/U) and simple schizophrenia (U-W/U-U-W-U-W-W-U-W-U) groups produced some differences, indicating that the difference of distribution of 10 finger pattern could determine clinical subtypes in schizophrenia. Currently, there is no enough data to describe the dermatoglypics pattern nature in subclinical types of schizophrenia.

In some studies, it noted that the total finger ridge count was decreased in schizophrenic patients as compared to control population [13,32]. There is no significant difference observed in our study groups. It might be related with relatively higher amount of loop and whorl in the schizophrenic and control groups.

It was previously reported that A-B ridge count was a main indicator to describe dermatoglypics characteristic nature of schizophrenia [33,34]. In our study, the reduced A-B ridge count was observed in schizophrenic as compared to control groups. The highest A-B ridge count (79.5±10.9) was detected in catatonic schizophrenia subclinical group and it was much higher than that in control and other subclinical groups. Hence, it seemed that the reduced A-B ridge count is more efficient marker to diagnose the schizophrenia and the high A-B ridge count is specific to recognize the catatonic schizophrenia among other types of schizophrenia.

The pattern intensity index represents the number of triradii on all ten fingers and it varies by race and ethnicity in the world [35–37]. The highest pattern intensity index is 16.65 in Ellice Islands and the lowest pattern intensity index is 9.97 in Bostwana. The average of pattern intensity index is evaluated 13.5 in the world. In our study, the pattern intensity index in schizophrenic patient group was somewhat lower than that in control group. The index in our study groups was detected higher in comparison with average of pattern intensity index in the world. More interestingly, the highest value of pattern intensity index was detected in catatonic schizophrenia group. In our study, Dankmeijer’s index in schizophrenic group was relatively higher than that in control group. Vashist et al also confirmed that the Dankmeijer’s index was higher in mental retardation [38]. The Dankmeijer’s index also exhibited sex and subclinical type differencesin the patient group.

The angles of “atd”, “tda” and “dat” were carefully evaluated to determine their nature properties related to schizophrenia. In the current study, no significant difference of the palmar triradii-related angles was produced in the schizophrenic patient and control groups. Birsen et al reported that a significant decrease of atd angle was observed in schizophrenic patients [19]. Importantly, strong significant differences on angles of “atd”, “tda” and “dat” were observed in subclinical groups as compared to control group.

Our results showed that the characteristics of dermatoglypics are crucial to differentiate the schizophrenia as compared to people without mental disorders, especially differentiation of clinical subtypes of schizophrenia. In the current study, it has considered that the main markers to describe the dermatoglypic nature of schizophrenia are the arch pattern type, more whorl distribution on all 10 fingers, a reduced A-B ridge count and lower pattern intensity index. In clinical subtypes of schizophrenia, the differences on dermatoglypic properties are more useful to differentiate them. More importantly, the catatonic schizophrenia had more different properties on fingerprint and palmar print patterns in comparison with other clinical subtypes of schizophrenia, which could be explained with the role of genetic factors in development of the catatonic schizophrenia.

## Limitation of the study

The relationship between dermatoglypic characteristics and schizophrenia might have some important outcome. In this study, we aimed to reveal the specified finger and palmar print patterns in schizophrenic subtypes. We have obtained some positive results from the study. In catatonic schizophrenia group, it had more differences on fingerprint and palmar print patterns in comparison with other clinical subtypes of schizophrenia. However, the sample size (n = 6) in the catatonic schizophrenia was small due to rare incidence among all schizophrenia subtypes. Thus, it has been considered that the sample size might influence the positive results of relationship between the dermatoglypic characteristics and schizophrenia subtypes in present study.

## Conclusion

This comparative study on schizophrenia-related dermatoglypic characteristics has found some specific markers of dermatoglypics for schizophrenia and its clinical types. Firstly, it was observed that the arch pattern type in the right ring finger was detected as a specific marker in schizophrenic patients. Then, the universal distribution in catatonic schizophrenia (W/U-W-W/U-W-W-W-W-W-W-W/U) and simple schizophrenia (U-W/U-U-W-U-W-W-U-W-U) groups was seemed to be a useful indicator to differentiate the clinical subtypes. Subsequently, a reduced A-B ridge count in schizophrenic patient group and significantly higher A-B ridge count in catatonic schizophrenia were found, suggesting that they can be used as a promising indicator in schizophrenia diagnosis. Our study also revealed that the catatonic schizophrenia had more mysterious patterns on dermatoglypic characteristics such as more whorl pattern types, high A-B ridge count and high pattern intensity index. Taking together, these results showed that the dermatoglypic characteristics might be a valuable tool to describe the nature of schizophrenia and its clinical subtypes and further studies are needed in clinical application.

## Supporting information

S1 File Images for fingerprint and palmar print evaluation in control group. (DOCX)

S2 File Images for fingerprint and palmar print evaluation in patient group. (DOCX)
