# Supplementary material for: Main characteristics of dermatoglypics associated with schizophrenia and its clinical subtypes
Source: PLoS One. 2021 Jun 10;16(6):e0252831. doi: 10.1371/journal.pone.0252831 (PMC8191880; doi:10.1371/journal.pone.0252831)
Supplement: S1 File — (DOCX) [file pone.0252831.s001.docx]

**Data of dermatoglypic characteristics**

**Control group**

1. Participant ID: M103

Sex: Female

Age: 50

DS: Sanus


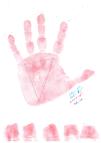

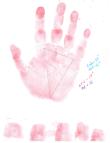


**Figure 1.** Fingerprint and palmar print.

2. Participant ID: M104

Sex: Female

Age: 51

DS: Sanus


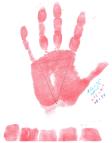

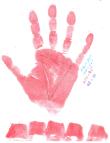


**Figure 2.** Fingerprint and palmar print.

3. Participant ID: M105

Sex: Female

Age: 41

DS: Sanus


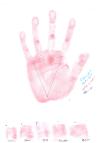

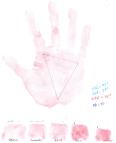


**Figure 3.** Fingerprint and palmar print.

4. Participant ID: M106

Sex: Female

Age: 56

DS: Sanus


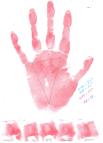

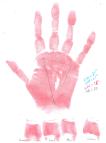


**Figure 4.** Fingerprint and palmar print.

5. Participant ID: M107

Sex: Female

Age: 40

DS: Sanus


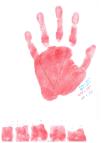

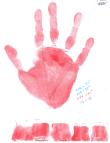


**Figure 5.** Fingerprint and palmar print.

6. Participant ID: M108

Sex: Female

Age: 43

DS: Sanus


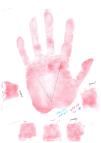

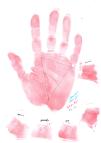


**Figure 6.** Fingerprint and palmar print.

7. Participant ID: M109

Sex: Female

Age: 38

DS: Sanus


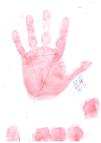

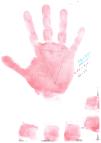


**Figure 7.** Fingerprint and palmar print.

8. Participant ID: M111

Sex: Male

Age: 38

DS: Sanus


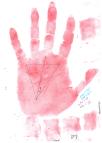

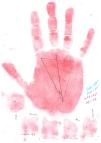


**Figure 8.** Fingerprint and palmar print.

9. Participant ID: M112

Sex: Male

Age: 51

DS: Sanus


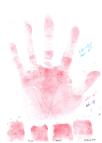

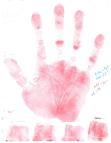


**Figure 9.** Fingerprint and palmar print.

10. Participant ID: M114

Sex: Female

Age: 43

DS: Sanus


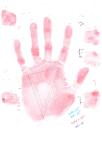

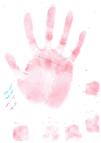


**Figure 10.** Fingerprint and palmar print.

11. Participant ID: M115

Sex: Female

Age: 52

DS: Sanus


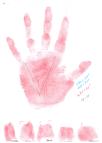

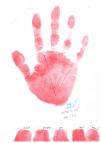


**Figure 11.** Fingerprint and palmar print.

12. Participant ID: M116

Sex: Male

Age: 56

DS: Sanus


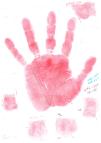

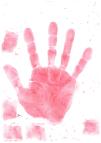


**Figure 12.** Fingerprint and palmar print.

13. Participant ID: M118

Sex: Male

Age: 57

DS: Sanus


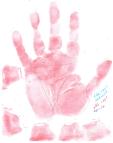

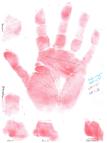


**Figure 13.** Fingerprint and palmar print.

14. Participant ID: M119

Sex: Female

Age: 42

DS: Sanus


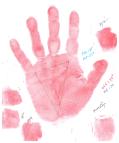

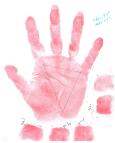


**Figure 14.** Fingerprint and palmar print.

15. Participant ID: M120

Sex: Female

Age: 33

DS: Sanus


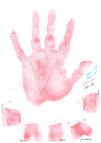

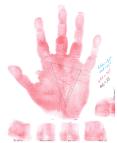


**Figure 15.** Fingerprint and palmar print.

16. Participant ID: M121

Sex: Male

Age: 33

DS: Sanus


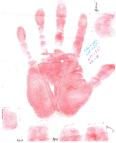

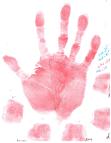


**Figure 16.** Fingerprint and palmar print.

17. Participant ID: M122

Sex: Female

Age: 32

DS: Sanus


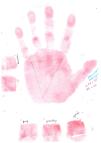

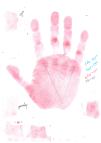


**Figure 17.** Fingerprint and palmar print.

18. Participant ID: M123

Sex: Female

Age: 28

DS: Sanus


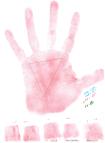

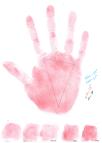


**Figure 18.** Fingerprint and palmar print.

19. Participant ID: M124

Sex: Female

Age: 26

DS: Sanus


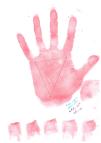

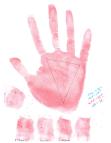


**Figure 19.** Fingerprint and palmar print.

20. Participant ID: M126

Sex: Female

Age: 38

DS: Sanus


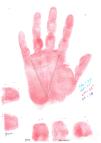

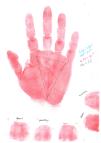


**Figure 20.** Fingerprint and palmar print.

21. Participant ID: M128

Sex: Female

Age: 39

DS: Sanus


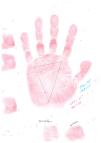

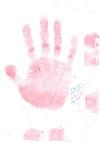


**Figure 21.** Fingerprint and palmar print.

22. Participant ID: M129

Sex: Female

Age: 36

DS: Sanus


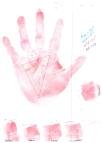

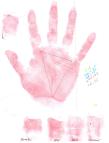


**Figure 22.** Fingerprint and palmar print.

23. Participant ID: M130

Sex: Female

Age: 36

DS: Sanus


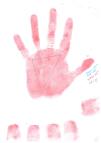

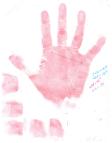


**Figure 23.** Fingerprint and palmar print.

24. Participant ID: M131

Sex: Male

Age: 36

DS: Sanus


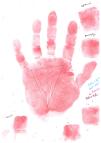

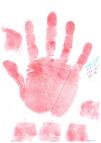


**Figure 24.** Fingerprint and palmar print.

25. Participant ID: M132

Sex: Female

Age: 33

DS: Sanus


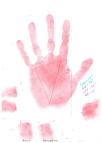

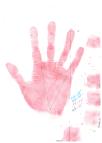


**Figure 25.** Fingerprint and palmar print.

26. Participant ID: M134

Sex: Female

Age: 46

DS: Sanus


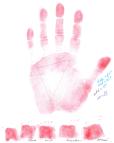

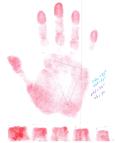


**Figure 26.** Fingerprint and palmar print.

27. Participant ID: M135

Sex: Male

Age: 27

DS: Sanus


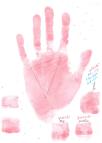

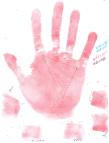


**Figure 27.** Fingerprint and palmar print.

28. Participant ID: M136

Sex: Male

Age: 31

DS: Sanus


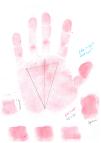

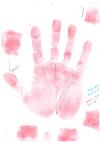


**Figure 28.** Fingerprint and palmar print.

29. Participant ID: M137

Sex: Male

Age: 25

DS: Sanus


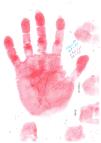

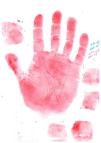


**Figure 29.** Fingerprint and palmar print.

30. Participant ID: M138

Sex: Male

Age: 26

DS: Sanus


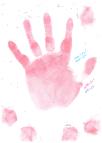

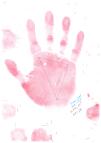


**Figure 30.** Fingerprint and palmar print.

31. Participant ID: M139

Sex: Male

Age: 32

DS: Sanus


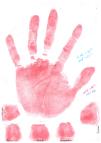

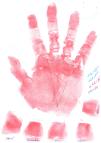


**Figure 31.** Fingerprint and palmar print.

32. Participant ID: M142

Sex: Female

Age: 37

DS: Sanus


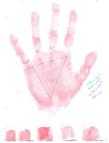

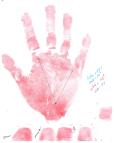


**Figure 32.** Fingerprint and palmar print.

33. Participant ID: M143

Sex: Male

Age: 22

DS: Sanus


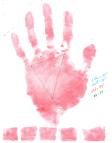

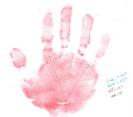

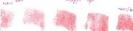

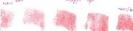


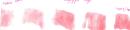


**Figure 33.** Fingerprint and palmar print.

34. Participant ID: M144

Sex: Male

Age: 24

DS: Sanus


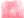

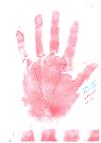

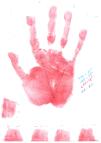


**Figure 34.** Fingerprint and palmar print.

35. Participant ID: M145

Sex: Male

Age: 22

DS: Sanus


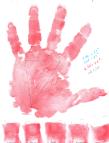

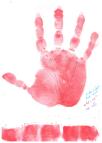


**Figure 35.** Fingerprint and palmar print.

36. Participant ID: M146

Sex: Male

Age: 21

DS: Sanus


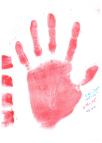

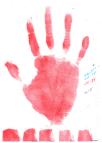


**Figure 36.** Fingerprint and palmar print.

37. Participant ID: M147

Sex: Male

Age: 22

DS: Sanus


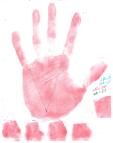

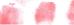

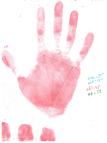


**Figure 37.** Fingerprint and palmar print.

38. Participant ID: M148

Sex: Male

Age: 27

DS: Sanus


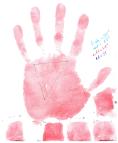

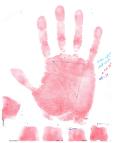


**Figure 38.** Fingerprint and palmar print.

39. Participant ID:M149

Sex: Male

Age: 22

DS: Sanus


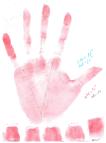

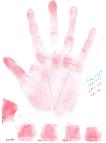


**Figure 39.** Fingerprint and palmar print.

40. Participant ID: M150

Sex: Male

Age: 28

DS: Sanus


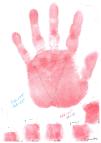

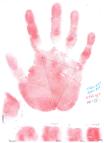


**Figure 40.** Fingerprint and palmar print.

41. Participant ID: M151

Sex: Male

Age: 22

DS: Sanus


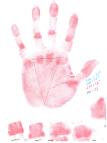

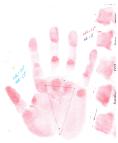


**Figure 41.** Fingerprint and palmar print.

42. Participant ID: M152

Sex: Male

Age: 22

DS: Sanus


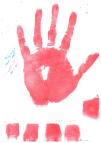

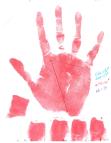


**Figure 42.** Fingerprint and palmar print.

43. Participant ID: M153

Sex: Male

Age: 23

DS: Sanus


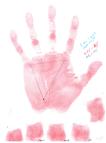

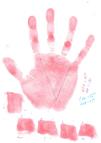


**Figure 43.** Fingerprint and palmar print.

44. Participant ID: M154

Sex: Male

Age: 21

DS: Sanus


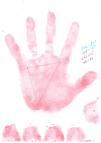

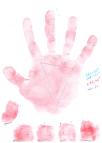


**Figure 44.** Fingerprint and palmar print.

45. Participant ID: M155

Sex: Male

Age: 29

DS: Sanus


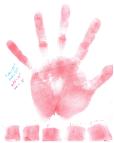

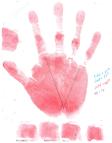


**Figure 45.** Fingerprint and palmar print.

46. Participant ID: M156

Sex: Male

Age: 27

DS: Sanus


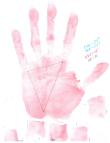

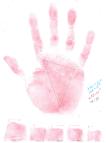


**Figure 46.** Fingerprint and palmar print.

47. Participant ID: M157

Sex: Male

Age: 24

DS: Sanus


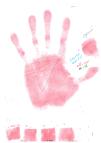

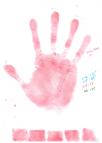


**Figure 47.** Fingerprint and palmar print.

48. Participant ID: M158

Sex: Male

Age: 32

DS: Sanus


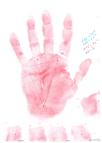


**Figure 48.** Fingerprint and palmar print.

49. Participant ID: M159

Sex: Male

Age: 33

DS: Sanus

**Figure 49.** Fingerprint and palmar print.

50. Participant ID: M160

Sex: Male

Age: 34

DS: Sanus

**Figure 50.** Fingerprint and palmar print.

51. Participant ID: M161

Sex: Male

Age: 34

DS: Sanus

**Figure 51.** Fingerprint and palmar print.

52. Participant ID: M162

Sex: Male

Age: 32

DS: Sanus

**Figure 52.** Fingerprint and palmar print.

53. Participant ID: M163

Sex: Male

Age: 31

DS: Sanus

**Figure 53.** Fingerprint and palmar print.

54. Participant ID: M164

Sex: Male

Age: 37

DS: Sanus

**Figure 54.** Fingerprint and palmar print.

55. Participant ID: M165

Sex: Male

Age: 34

DS: Sanus

**Figure 55.** Fingerprint and palmar print.

56. Participant ID: M166

Sex: Male

Age: 30

DS: Sanus

**Figure 56.** Fingerprint and palmar print.

57. Participant ID: M167

Sex: Male

Age: 33

DS: Sanus

**Figure 57.** Fingerprint and palmar print.

58. Participant ID: M168

Sex: Male

Age: 36

DS: Sanus

**Figure 58.** Fingerprint and palmar print.

59. Participant ID: M169

Sex: Male

Age: 44

DS: Sanus

**Figure 59.** Fingerprint and palmar print.

60. Participant ID:M170

Sex: Male

Age: 43

DS: Sanus

**Figure 60.** Fingerprint and palmar print.

61. Participant ID: M171

Sex: Male

Age: 41

DS: Sanus

**Figure 61.** Fingerprint and palmar print.

62. Participant ID: M172

Sex: Male

Age: 47

DS: Sanus

**Figure 62.** Fingerprint and palmar print.

63. Participant ID: M173

Sex: Male

Age: 41

DS: Sanus

**Figure 63.** Fingerprint and palmar print.

64. Participant ID: M174

Sex: Male

Age: 42

DS: Sanus

**Figure 64.** Fingerprint and palmar print.

65. Participant ID: M175

Sex: Male

Age: 43

DS: Sanus

**Figure 65.** Fingerprint and palmar print.

66. Participant ID: M177

Sex: Male

Age: 44

DS: Sanus

**Figure 66.** Fingerprint and palmar print.

67. Participant ID: M178

Sex: Male

Age: 47

DS: Sanus

**Figure 67.** Fingerprint and palmar print.

68. Participant ID: M179

Sex: Male

Age: 40

DS: Sanus

**Figure 68.** Fingerprint and palmar print.

69. Participant ID: M180

Sex: Male

Age: 41

DS: Sanus

**Figure 69.** Fingerprint and palmar print.

70. Participant ID: M181

Sex: Male

Age: 46

DS: Sanus

**Figure 70.** Fingerprint and palmar print.

71. Participant ID: M182

Sex: Male

Age: 40

DS: Sanus

**Figure 71.** Fingerprint and palmar print.

72. Participant ID: M183

Sex: Male

Age: 43

DS: Sanus

**Figure 72.** Fingerprint and palmar print.

73. Participant ID: M184

Sex: Male

Age: 53

DS: Sanus

**Figure 73.** Fingerprint and palmar print.

74. Participant ID: M185

Sex: Male

Age: 58

DS: Sanus

**Figure 74.** Fingerprint and palmar print.

75. Participant ID: M186

Sex: Male

Age: 53

DS: Sanus

**Figure 75.** Fingerprint and palmar print.

76. Participant ID: M187

Sex: Male

Age: 51

DS: Sanus

**Figure 76.** Fingerprint and palmar print.

77. Participant ID: M188

Sex: Male

Age: 51

DS: Sanus

**Figure 77.** Fingerprint and palmar print.

78. Participant ID: M189

Sex: Male

Age:55

DS: Sanus

**Figure 78.** Fingerprint and palmar print.

79. Participant ID: M190

Sex: Male

Age: 55

DS: Sanus

**Figure 79.** Fingerprint and palmar print.

80. Participant ID: M191

Sex: Male

Age: 55

DS: Sanus

**Figure 80.** Fingerprint and palmar print.

81. Participant ID: M192

Sex: Male

Age: 55

DS: Sanus

**Figure 81.** Fingerprint and palmar print.

82. Participant ID: M193

Sex: Male

Age: 55

DS: Sanus

**Figure 82.** Fingerprint and palmar print.

83. Participant ID: M194

Sex: Male

Age: 56

DS: Sanus

**Figure 83.** Fingerprint and palmar print.

84. Participant ID: M195

Sex: Male

Age: 56

DS: Sanus

**Figure 84.** Fingerprint and palmar print.

85. Participant ID: M196

Sex: Male

Age: 55

DS: Sanus

**Figure 85.** Fingerprint and palmar print.

86. Participant ID: M197

Sex: Male

Age: 50

DS: Sanus

**Figure 86.** Fingerprint and palmar print.

87. Participant ID: M198

Sex: Male

Age: 57

DS: Sanus

**Figure 87.** Fingerprint and palmar print.

88. Participant ID: M199

Sex: Male

Age: 60

DS: Sanus

**Figure 88.** Fingerprint and palmar print.

89. Participant ID: M200

Sex: Male

Age: 63

DS: Sanus

**Figure 89.** Fingerprint and palmar print.

90. Participant ID: M201

Sex: Male

Age: 63

DS: Sanus

**Figure 90.** Fingerprint and palmar print.

91. Participant ID: M202

Sex: Female

Age: 21

DS: Sanus

**Figure 91.** Fingerprint and palmar print.

92. Participant ID: M203

Sex: Female

Age: 23

DS: Sanus

**Figure 92.** Fingerprint and palmar print.

93. Participant ID: M204

Sex: Female

Age: 23

DS: Sanus

**Figure 93.** Fingerprint and palmar print.

94. Participant ID: M205

Sex: Female

Age: 22

DS: Sanus

**Figure 94.** Fingerprint and palmar print.

95. Participant ID: M206

Sex: Female

Age: 22

DS: Sanus

**Figure 95.** Fingerprint and palmar print.

96. Participant ID: M207

Sex: Female

Age: 22

DS: Sanus

**Figure 96.** Fingerprint and palmar print.

97. Participant ID: M208

Sex: Female

Age: 23

DS: Sanus

**Figure 97.** Fingerprint and palmar print.

98. Participant ID: M209

Sex: Female

Age: 23

DS: Sanus

**Figure 98.** Fingerprint and palmar print.

99. Participant ID: M210

Sex: Female

Age: 22

DS: Sanus

**Figure 99.** Fingerprint and palmar print.

100. Participant ID: M211

Sex: Female

Age: 21

DS: Sanus

**Figure 100.** Fingerprint and palmar print.

101. Participant ID: M212

Sex: Female

Age: 22

DS: Sanus

**Figure 101.** Fingerprint and palmar print.

102. Participant ID: M213

Sex: Female

Age: 22

DS: Sanus

**Figure 102.** Fingerprint and palmar print.

103. Participant ID: M214

Sex: Female

Age: 22

DS: Sanus

**Figure 103.** Fingerprint and palmar print.

104. Participant ID: M215

Sex: Female

Age: 23

DS: Sanus

**Figure 104.** Fingerprint and palmar print.

105. Participant ID: M216

Sex: Female

Age: 29

DS: Sanus

**Figure 105.** Fingerprint and palmar print.

106. Participant ID: M217

Sex: Female

Age: 32

DS: Sanus

**Figure 106.** Fingerprint and palmar print.

107. Participant ID: M218

Sex: Female

Age: 30

DS: Sanus

**Figure 107.** Fingerprint and palmar print.

108. Participant ID: M219

Sex: Female

Age: 39

DS: Sanus

**Figure 108.** Fingerprint and palmar print.

109. Participant ID: M220

Sex: Female

Age: 38

DS: Sanus

**Figure 109.** Fingerprint and palmar print.

110. Participant ID: M221

Sex: Female

Age: 32

DS: Sanus

**Figure 110.** Fingerprint and palmar print.

111. Participant ID: M222

Sex: Female

Age: 30

DS: Sanus

**Figure 111.** Fingerprint and palmar print.

112. Participant ID: M223

Sex: Female

Age: 36

DS: Sanus

**Figure 112.** Fingerprint and palmar print.

113. Participant ID: M224

Sex: Female

Age: 32

DS: Sanus

**Figure 113.** Fingerprint and palmar print.

114. Participant ID: M225

Sex: Female

Age: 49

DS: Sanus

**Figure 114.** Fingerprint and palmar print.

115. Participant ID: M226

Sex: Female

Age: 45

DS: Sanus

**Figure 115.** Fingerprint and palmar print.

116. Participant ID: M227

Sex: Female

Age: 48

DS: Sanus

**Figure 116.** Fingerprint and palmar print.

117. Participant ID: M228

Sex: Female

Age: 45

DS: Sanus

**Figure 117.** Fingerprint and palmar print.

118. Participant ID: M229

Sex: Female

Age: 40

DS: Sanus

**Figure 118.** Fingerprint and palmar print.

119. Participant ID: M230

Sex: Female

Age: 48

DS: Sanus

**Figure 119.** Fingerprint and palmar print.

120. Participant ID: M231

Sex: Female

Age: 41

DS: Sanus

**Figure 120.** Fingerprint and palmar print.

121. Participant ID: M232

Sex: Female

Age: 46

DS: Sanus

**Figure 121.** Fingerprint and palmar print.

122. Participant ID: M233

Sex: Female

Age: 46

DS: Sanus

**Figure 122.** Fingerprint and palmar print.

123. Participant ID: M234

Sex: Female

Age: 41

DS: Sanus

**Figure 123.** Fingerprint and palmar print.

124. Participant ID: M235

Sex: Female

Age: 49

DS: Sanus

**Figure 124.** Fingerprint and palmar print.

125. Participant ID: M236

Sex: Female

Age: 43

DS: Sanus

**Figure 125.** Fingerprint and palmar print.

126. Participant ID: M237

Sex: Female

Age: 52

DS: Sanus

**Figure 126.** Fingerprint and palmar print.

127. Participant ID: M238

Sex: Female

Age: 55

DS: Sanus

**Figure 127.** Fingerprint and palmar print.

128. Participant ID: M239

Sex: Female

Age: 56

DS: Sanus

**Figure 128.** Fingerprint and palmar print.

129. Participant ID: M240

Sex: Female

Age: 55

DS: Sanus

**Figure 129.** Fingerprint and palmar print.

130. Participant ID: M241

Sex: Female

Age: 58

DS: Sanus

**Figure 130.** Fingerprint and palmar print.

131. Participant ID: M242

Sex: Female

Age: 57

DS: Sanus

**Figure 131.** Fingerprint and palmar print.

132. Participant ID: M243

Sex: Female

Age: 56

DS: Sanus

**Figure 132.** Fingerprint and palmar print.

133. Participant ID: M244

Sex: Female

Age: 55

DS: Sanus

**Figure 133.** Fingerprint and palmar print.

134. Participant ID: M245

Sex: Female

Age: 53

DS: Sanus

**Figure 134.** Fingerprint and palmar print.

135. Participant ID: M246

Sex: Female

Age: 55

DS: Sanus

**Figure 135.** Fingerprint and palmar print.

136. Participant ID: M247

Sex: Female

Age: 56

DS: Sanus

**Figure 136.** Fingerprint and palmar print.

137. Participant ID: M248

Sex: Female

Age: 57

DS: Sanus

**Figure 137.** Fingerprint and palmar print.

138. Participant ID: M249

Sex: Female

Age: 55

DS: Sanus

**Figure 138.** Fingerprint and palmar print.

139. Participant ID: M250

Sex: Female

Age: 65

DS: Sanus

**Figure 139.** Fingerprint and palmar print.

140. Participant ID: M251

Sex: Female

Age: 62

DS: Sanus

**Figure 140.** Fingerprint and palmar print.

141. Participant ID: M252

Sex: Female

Age: 66

DS: Sanus

**Figure 141.** Fingerprint and palmar print.
