# Supplementary material for: Main characteristics of dermatoglypics associated with schizophrenia and its clinical subtypes
Source: PLoS One. 2021 Jun 10;16(6):e0252831. doi: 10.1371/journal.pone.0252831 (PMC8191880; doi:10.1371/journal.pone.0252831)
Supplement: S2 File — (DOCX) [file pone.0252831.s002.docx]

**Data of dermatoglypic characteristics**

**Patient group**

1. Patient ID: M001

Sex: Female

Age: 39

DS: F2 Schizophrenia Clinical subtype: Paranoid


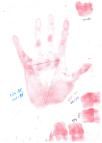

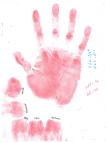


**Figure 1.** Fingerprint and palmar print.

2. Patient ID: M003

Sex: Female

Age: 34

DS: F2 Schizophrenia Clinical subtype: Paranoid


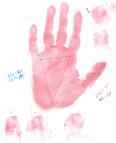

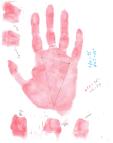


**Figure 2.** Fingerprint and palmar print.

3. Patient ID: M005

Sex: Female

Age: 45

DS: F2 Schizophrenia Clinical subtype: Catatonic


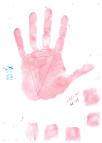

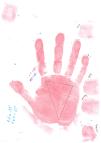


**Figure 3.** Fingerprint and palmar print.

4. Patient ID: M007

Sex: Female

Age: 23

DS: F2 Schizophrenia Clinical subtype: Paranoid


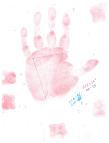

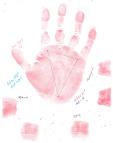


**Figure 4.** Fingerprint and palmar print.

5. Patient ID: M008

Sex: Male

Age: 32

DS: F2 Schizophrenia Clinical subtype: Paranoid


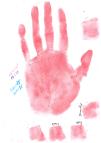

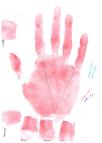


**Figure 5.** Fingerprint and palmar print.

6. Patient ID: M009

Sex: Female

Age: 30

DS: F2 Schizophrenia Clinical subtype: Residual


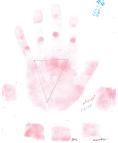

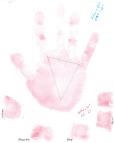


**Figure 6.** Fingerprint and palmar print.

7. Patient ID: M010

Sex: Female

Age: 52

DS: F2 Schizophrenia Clinical subtype: Paranoid


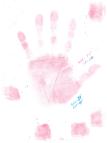

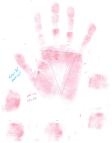


**Figure 7.** Fingerprint and palmar print.

8. Patient ID: M011

Sex: Female

Age: 48

DS: F2 Schizophrenia Clinical subtype: Simple


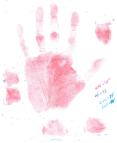

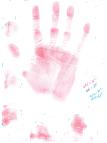


**Figure 8.** Fingerprint and palmar print.

9. Patient ID: M012

Sex: Male

Age: 37

DS: F2 Schizophrenia Clinical subtype: Paranoid


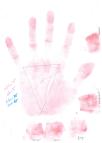

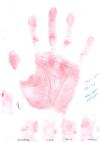


**Figure 9.** Fingerprint and palmar print.

10. Patient ID: M013

Sex: Female

Age: 36

DS: F2 Schizophrenia Clinical subtype: Paranoid


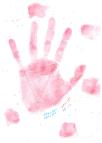

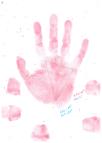


**Figure 10.** Fingerprint and palmar print.

11. Patient ID: M014

Sex: Male

Age: 31

DS: F2 Schizophrenia Clinical subtype: Paranoid


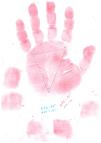

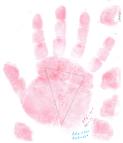


**Figure 11.** Fingerprint and palmar print.

12. Patient ID: M015

Sex: Male

Age: 36

DS: F2 Schizophrenia Clinical subtype: Paranoid


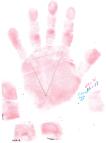

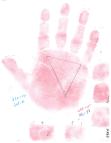


**Figure 12.** Fingerprint and palmar print.

13. Patient ID: M016

Sex: Female

Age: 36

DS: F2 Schizophrenia Clinical subtype: Paranoid


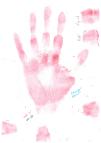

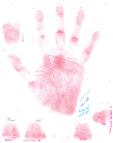


**Figure 13.** Fingerprint and palmar print.

14. Patient ID: M020

Sex: Female

Age: 35

DS: F2 Schizophrenia Clinical subtype: Simple


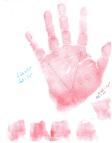

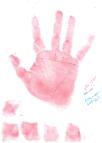


**Figure 14.** Fingerprint and palmar print.

15. Patient ID: M021

Sex: Female

Age: 44

DS: F2 Schizophrenia Clinical subtype: Paranoid


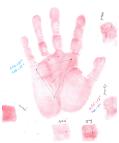

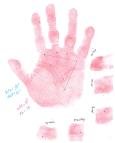


**Figure 15.** Fingerprint and palmar print.

16. Patient ID: M022

Sex: Female

Age: 35

DS: F2 Schizophrenia Clinical subtype: Paranoid


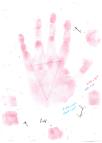

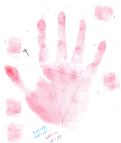


**Figure 16.** Fingerprint and palmar print.

17. Patient ID: M023

Sex: Female

Age: 27

DS: F2 Schizophrenia Clinical subtype: Paranoid


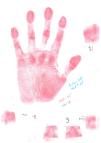

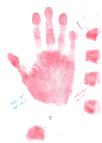


**Figure 17.** Fingerprint and palmar print.

18. Patient ID: M024

Sex: Male

Age: 46

DS: F2 Schizophrenia Clinical subtype: Residual


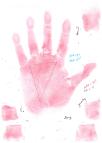

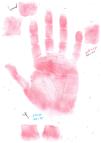


**Figure 18.** Fingerprint and palmar print.

19. Patient ID: M025

Sex: Male

Age: 28

DS: F2 Schizophrenia Clinical subtype: Paranoid


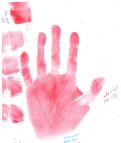

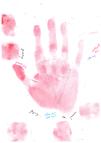


**Figure 19.** Fingerprint and palmar print.

20. Patient ID: M026

Sex: Female

Age: 50

DS: F2 Schizophrenia Clinical subtype: Residual


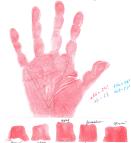

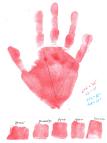


**Figure 20.** Fingerprint and palmar print.

21. Patient ID: M027

Sex: Female

Age: 54

DS: F2 Schizophrenia Clinical subtype: Residual


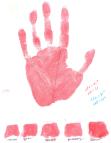

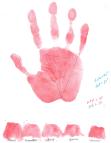


**Figure 21.** Fingerprint and palmar print.

22. Patient ID: M028

Sex: Female

Age: 53

DS: F2 Schizophrenia Clinical subtype: Residual


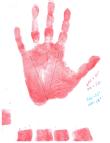

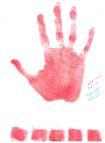


**Figure 22.** Fingerprint and palmar print.

23. Patient ID: M029

Sex: Female

Age: 51

DS: F2 Schizophrenia Clinical subtype: Residual


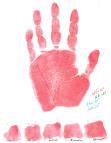

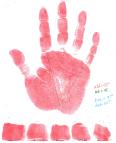


**Figure 23.** Fingerprint and palmar print.

24. Patient ID: M030

Sex: Male

Age:51

DS: F2 Schizophrenia Clinical subtype: Simple


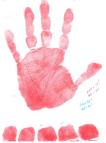

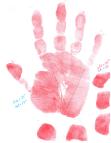


**Figure 24.** Fingerprint and palmar print.

25. Patient ID: M031

Sex: Male

Age: 44

DS: F2 Schizophrenia Clinical subtype: Paranoid


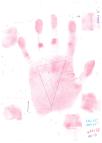

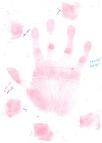


**Figure 25.** Fingerprint and palmar print.

26. Patient ID: M032

Sex: Male

Age: 26

DS: F2 Schizophrenia Clinical subtype: Paranoid


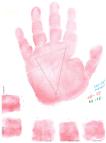

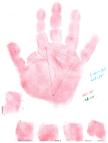


**Figure 26.** Fingerprint and palmar print.

27. Patient ID: M033

Sex: Male

Age: 52

DS: F2 Schizophrenia Clinical subtype: Paranoid


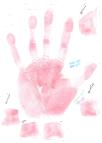

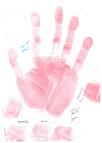


**Figure 27.** Fingerprint and palmar print.

28. Patient ID: M034

Sex: Male

Age: 51

DS: F2 Schizophrenia Clinical subtype: Simple


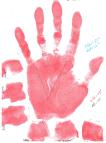

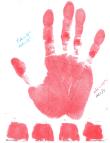


**Figure 28.** Fingerprint and palmar print.

29. Patient ID: M035

Sex: Male

Age: 30

DS: F2 Schizophrenia Clinical subtype: Paranoid


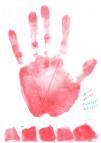

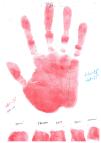


**Figure 29.** Fingerprint and palmar print.

30. Patient ID: M036

Sex: Female

Age: 29

DS: F2 Schizophrenia Clinical subtype: Paranoid


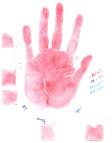

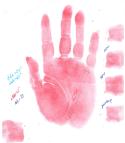


**Figure 30.** Fingerprint and palmar print.

31. Patient ID: M037

Sex: Female

Age: 42

DS: F2 Schizophrenia Clinical subtype: Residual


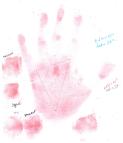

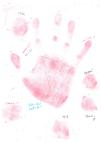


**Figure 31.** Fingerprint and palmar print.

32. Patient ID: M038

Sex: Female

Age: 37

DS: F2 Schizophrenia Clinical subtype: Paranoid


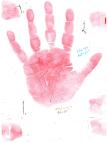

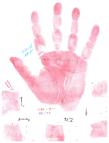


**Figure 32.** Fingerprint and palmar print.

33. Patient ID: M042

Sex: Female

Age: 52

DS: F2 Schizophrenia Clinical subtype: Catatonic


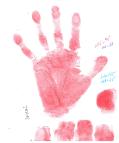

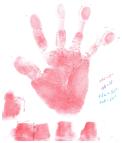


**Figure 33.** Fingerprint and palmar print.

34. Patient ID: M043

Sex: Male

Age: 20

DS: F2 Schizophrenia Clinical subtype: Paranoid


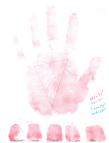

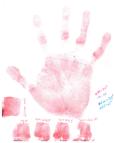


**Figure 34.** Fingerprint and palmar print.

35. Patient ID: M044

Sex: Male

Age: 24

DS: F2 Schizophrenia Clinical subtype: Paranoid


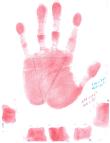

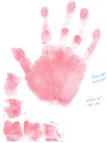


**Figure 35.** Fingerprint and palmar print.

36. Patient ID: M045

Sex: Male

Age: 23

DS: F2 Schizophrenia Clinical subtype: Residual


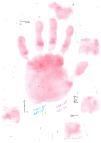

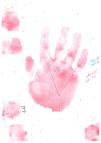


**Figure 36.** Fingerprint and palmar print.

37. Patient ID: M046

Sex: Male

Age: 28

DS: F2 Schizophrenia Clinical subtype: Residual


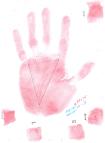

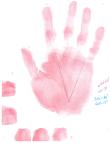


**Figure 37.** Fingerprint and palmar print.

38. Patient ID: M047

Sex: Male

Age: 26

DS: F2 Schizophrenia Clinical subtype: Paranoid


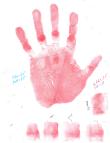

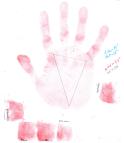


**Figure 38.** Fingerprint and palmar print.

39. Patient ID: M048

Sex: Male

Age: 22

DS: F2 Schizophrenia Clinical subtype: Paranoid


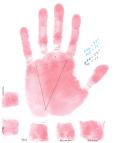

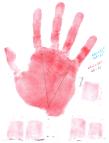


**Figure 39.** Fingerprint and palmar print.

40. Patient ID: M049

Sex: Male

Age: 21

DS: F2 Schizophrenia Clinical subtype: Simple


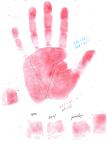

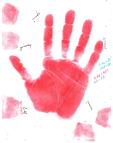


**Figure 40.** Fingerprint and palmar print.

41. Patient ID: M050

Sex: Male

Age: 32

DS: F2 Schizophrenia Clinical subtype: Residual


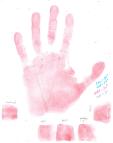

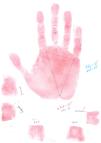


**Figure 41.** Fingerprint and palmar print.

42. Patient ID: M051

Sex: Male

Age: 35

DS: F2 Schizophrenia Clinical subtype: Paranoid


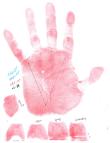

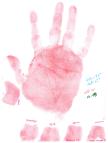


**Figure 42.** Fingerprint and palmar print.

43. Patient ID: M052

Sex: Male

Age: 36

DS: F2 Schizophrenia Clinical subtype: Paranoid


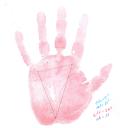

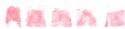

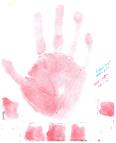


**Figure 43.** Fingerprint and palmar print.

44. Patient ID: M053

Sex: Male

Age: 31

DS: F2 Schizophrenia Clinical subtype: Simple


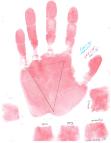

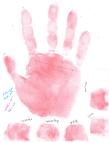


**Figure 44.** Fingerprint and palmar print.

45. Patient ID: M054

Sex: Male

Age: 38

DS: F2 Schizophrenia Clinical subtype: Paranoid


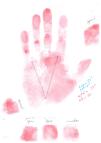

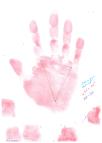


**Figure 45.** Fingerprint and palmar print.

46. Patient ID: M055

Sex: Male

Age: 32

DS: F2 Schizophrenia Clinical subtype: Paranoid


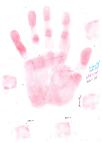

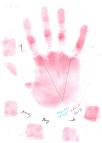


**Figure 46.** Fingerprint and palmar print.

47. Patient ID: M056

Sex: Male

Age: 33

DS: F2 Schizophrenia Clinical subtype: Catatonic


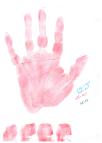

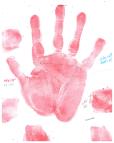


**Figure 47.** Fingerprint and palmar print.

48. Patient ID: M057

Sex: Male

Age: 36

DS: F2 Schizophrenia Clinical subtype: Paranoid


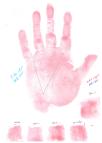

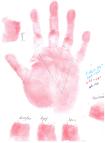


**Figure 48.** Fingerprint and palmar print.

49. Patient ID: M058

Sex: Male

Age: 45

DS: F2 Schizophrenia Clinical subtype: Paranoid


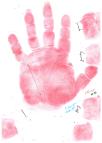

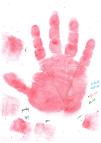


**Figure 49.** Fingerprint and palmar print.

50. Patient ID: M059

Sex: Male

Age: 42

DS: F2 Schizophrenia Clinical subtype: Residual


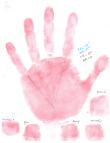


**Figure 50.** Fingerprint and palmar print.

51. Patient ID: M060

Sex: Male

Age: 43

DS: F2 Schizophrenia Clinical subtype: Paranoid

**Figure 51.** Fingerprint and palmar print.

52. Patient ID: M061

Sex: Male

Age: 45

DS: F2 Schizophrenia Clinical subtype: Residual

**Figure 52.** Fingerprint and palmar print.

53. Patient ID: M062

Sex: Male

Age: 46

DS: F2 Schizophrenia Clinical subtype: Residual

**Figure 53.** Fingerprint and palmar print.

54. Patient ID: M063

Sex: Female

Age: 43

DS: F2 Schizophrenia Clinical subtype: Paranoid

**Figure 54.** Fingerprint and palmar print.

55. Patient ID: M064

Sex: Male

Age: 46

DS: F2 Schizophrenia Clinical subtype: Simple

**Figure 55.** Fingerprint and palmar print.

56. Patient ID: M065

Sex: Male

Age: 40

DS: F2 Schizophrenia Clinical subtype: Residual

**Figure 56.** Fingerprint and palmar print.

57. Patient ID: M066

Sex: Male

Age: 58

DS:F2 Schizophrenia Clinical subtype: Cataonic

**Figure 57.** Fingerprint and palmar print.

58. Patient ID: M067

Sex: Male

Age: 59

DS: F2 Schizophrenia Clinical subtype: Residual

**Figure 58.** Fingerprint and palmar print.

59. Patient ID: M068

Sex: Male

Age: 51

DS: F2 Schizophrenia Clinical subtype: Residual

**Figure 59.** Fingerprint and palmar print.

60. Patient ID: M069

Sex: Male

Age: 54

DS: F2 Schizophrenia Clinical subtype: Residual

**Figure 60.** Fingerprint and palmar print.

61. Patient ID: M070

Sex: Male

Age: 59

DS: F2 Schizophrenia Clinical subtype: Residual

**Figure 61.** Fingerprint and palmar print.

62. Patient ID: M071

Sex: Male

Age: 54

DS: F2 Schizophrenia Clinical subtype: Residual

**Figure 62.** Fingerprint and palmar print.

63. Patient ID: M072

Sex: Male

Age: 54

DS: F2 Schizophrenia Clinical subtype: Residual

**Figure 63.** Fingerprint and palmar print.

64. Patient ID: M073

Sex: Male

Age: 55

DS: F2 Schizophrenia Clinical subtype: Residual

**Figure 64.** Fingerprint and palmar print.

65. Patient ID: M074

Sex: Male

Age: 57

DS: F2 Schizophrenia Clinical subtype: Residual

**Figure 65.** Fingerprint and palmar print.

66. Patient ID: M075

Sex: Male

Age: 64

DS: F2 Schizophrenia Clinical subtype: Residual

**Figure 66.** Fingerprint and palmar print.

67. Patient ID: M076

Sex: Female

Age: 29

DS: F2 Schizophrenia Clinical subtype: Paranoid

**Figure 67.** Fingerprint and palmar print.

68. Patient ID: M077

Sex: Female

Age: 28

DS: F2 Schizophrenia Clinical subtype: Paranoid

**Figure 68.** Fingerprint and palmar print.

69. Patient ID: M078

Sex: Female

Age: 29

DS: F2 Schizophrenia Clinical subtype: Paranoid

**Figure 69.** Fingerprint and palmar print.

70. Patient ID: M079

Sex: Female

Age: 26

DS: F2 Schizophrenia Clinical subtype: Paranoid

**Figure 70.** Fingerprint and palmar print.

71. Patient ID: M080

Sex: Female

Age: 23

DS: F2 Schizophrenia Clinical subtype: Paranoid

**Figure 71.** Fingerprint and palmar print.

72. Patient ID: M081

Sex: Female

Age: 24

DS: F2 Schizophrenia Clinical subtype: Paranoid

**Figure 72.** Fingerprint and palmar print.

73. Patient ID: M082

Sex: Female

Age: 29

DS: F2 Schizophrenia Clinical subtype: Catatonic

**Figure 73.** Fingerprint and palmar print.

74. Patient ID: M083

Sex: Female

Age: 28

DS: F2 Schizophrenia Clinical subtype: Paranoid

**Figure 74.** Fingerprint and palmar print.

75. Patient ID: M084

Sex: Female

Age: 22

DS: F2 Schizophrenia Clinical subtype: Paranoid

**Figure 75.** Fingerprint and palmar print.

76. Patient ID: M085

Sex: Female

Age: 39

DS: F2 Schizophrenia Clinical subtype: Paranoid

**Figure 76.** Fingerprint and palmar print.

77. Patient ID: M086

Sex: Female

Age: 37

DS: F2 Schizophrenia Clinical subtype: Paranoid

**Figure 77.** Fingerprint and palmar print.

78. Patient ID: M087

Sex: Female

Age: 44

DS: F2 Schizophrenia Clinical subtype: Simple

**Figure 78.** Fingerprint and palmar print.

79. Patient ID: M088

Sex: Female

Age: 40

DS: F2 Schizophrenia Clinical subtype: Paranoid

**Figure 79.** Fingerprint and palmar print.

80. Patient ID: M089

Sex: Female

Age: 48

DS: F2 Schizophrenia Clinical subtype: Paranoid

**Figure 80.** Fingerprint and palmar print.

81. Patient ID: M090

Sex: Female

Age: 46

DS: F2 Schizophrenia Clinical subtype: Residual

**Figure 81.** Fingerprint and palmar print.

82. Patient ID: M091

Sex: Female

Age: 49

DS: F2 Schizophrenia Clinical subtype: Paranoid

**Figure 82.** Fingerprint and palmar print.

83. Patient ID: M092

Sex: Female

Age: 41

DS: F2 Schizophrenia Clinical subtype: Paranoid

**Figure 83.** Fingerprint and palmar print.

84. Patient ID: M093

Sex: Female

Age: 56

DS: F2 Schizophrenia Clinical subtype: Residual

**Figure 84.** Fingerprint and palmar print.

85. Patient ID: M094

Sex: Female

Age: 56

DS: F2 Schizophrenia Clinical subtype: Catatonic

**Figure 85.** Fingerprint and palmar print.

86. Patient ID: M095

Sex: Female

Age: 54

DS: F2 Schizophrenia Clinical subtype: Paranoid

**Figure 86.** Fingerprint and palmar print.

87. Patient ID: M096

Sex: Female

Age: 56

DS: F2 Schizophrenia Clinical subtype: Residual

**Figure 87.** Fingerprint and palmar print.

88. Patient ID: M097

Sex: Female

Age: 53

DS: F2 Schizophrenia Clinical subtype: Residual

**Figure 88.** Fingerprint and palmar print.

89. Patient ID: M098

Sex: Female

Age: 54

DS: F2 Schizophrenia Clinical subtype: Simple

**Figure 89.** Fingerprint and palmar print.

90. Patient ID: M099

Sex: Female

Age: 51

DS: F2 Schizophrenia Clinical subtype: Residual

**Figure 90.** Fingerprint and palmar print.

91. Patient ID: M100

Sex: Male

Age: 49

DS: F2 Schizophrenia Clinical subtype: Paranoid

**Figure 91.** Fingerprint and palmar print.

92. Patient ID: M101

Sex: Female

Age: 27

DS: F2 Schizophrenia Clinical subtype: Paranoid

**Figure 92.** Fingerprint and palmar print.

93. Patient ID: M102

Sex: Female

Age: 22

DS: F2 Schizophrenia Clinical subtype: Simple

**Figure 93.** Fingerprint and palmar print.
